# Health-related quality of life and its influencing factors among people with heart failure in Ethiopia: using the revised Wilson and Cleary model

**DOI:** 10.1038/s41598-023-47567-x

**Published:** 2023-11-19

**Authors:** Henok Mulugeta, Peter M. Sinclair, Amanda Wilson

**Affiliations:** 1 Department of Nursing, College of Health Science, Debre Markos University, Debre Markos, Amhara Region Ethiopia; 2 School of Nursing and Midwifery, Faculty of Health, University of Technology Sydney, Sydney, NSW Australia

**Keywords:** Cardiology, Health care

## Abstract

Heart failure is a challenging public health problem associated with poor health-related quality of life (HRQoL). Data on the quality of life of people with heart failure are limited in Ethiopia. This study aimed to assess the HRQoL and its influencing factors in people with heart failure in Ethiopia. A hospital-based, cross-sectional study design was conducted in the cardiac outpatient clinics of two tertiary-level hospitals in Addis Ababa, Ethiopia. Data were collected from people with heart failure who met the inclusion criteria using an interviewer-administered questionnaire. The HRQoL was measured using the Minnesota Living with Heart Failure Questionnaire (MLHFQ). A multiple linear regression model was fitted to identify factors that influenced HRQoL. All statistical analyses were conducted using STATA version 17 software. A total of 383 people with heart failure participated in the study. The mean age of the participants was 55 years. The MLHFQ score was 48.03±19.73, and 54% of participants had poor HRQoL. Multiple linear regression analysis revealed that age (β= 0.12, 95% CI 0.11, 0.28), diabetes mellitus comorbidity (β= 4.47, 95% CI 1.41, 7.54), social support score (β= − 1.48, 95% CI − 1.93, − 1.03), and depression score (β = 1.74, 95% CI 1.52, 1.96) were significant factors influencing overall HRQoL (p < 0.05). This study found that people in Ethiopia with heart failure had poor HRQoL, influenced by several factors. The findings can help health professionals identify appropriate interventions to improve the HRQoL of people with heart failure.

## Introduction

Heart failure (HF) is a chronic medical condition associated with high morbidity and mortality rates^1^. It is diagnosed in one to two of every 100 adults in the general population, but the actual prevalence is probably around 4%^2^. Its risk factors include age, coronary artery disease, rheumatic heart disease, hypertension, and diabetes mellitus. Heart failure is the most common type of cardiovascular disease (CVD) in Africa and has a great social and economic impact mainly affecting young individuals. Rheumatic heart disease is one of the most prevalent risk factors for HF, occurring in 14 % of people with HF in sub-Saharan Africa^3–5^. According to the international congestive heart failure study report, the highest annual mortality in people with HF was observed in Africa^6^. In Ethiopia, the in-hospital mortality due to HF is high^7^.

Health-related quality of life (HRQoL) is an individual’s perception of their physical, social, emotional, psychological, and mental functioning^8,9^. Measuring HRQoL is often used to assess the effectiveness of HF treatment and the course of the disease^10^. Living with HF is challenging as it has a poor prognosis and increased socioeconomic burden due to increased healthcare costs, debilitating symptoms such as dyspnoea, oedema, fatigue, and sleep disturbance, and frequent hospitalisations which collectively, significantly impact HRQoL. Poor HRQoL increases the risk of hospitalisation and death^11,12^.

Many factors influence the HRQoL of people with HF. The revised Wilson and Clearly model is a practical conceptual framework that incorporates the relationship between different health concepts to guide quality of life research^13^. According to this model, HRQoL is affected by individual characteristics, biological function, environmental characteristics, symptoms, functional status, and general health perceptions. Previous studies have identified multiple predictors of poor HRQoL including gender (female), age (young), marital status (single), income and educational levels (low), presence of depression, high New York Heart Association (NYHA) functional class, tobacco exposure, low ventricular ejection, history of hospitalisation, comorbidity, polypharmacy, poor medication adherence and duration of heart failure^14–20^. Identifying meaningful factors influencing HRQoL is critical to develop effective interventions to improve prognosis in this cohort^21^.

Improving symptoms and physical functioning are the major aims of heart failure management. Effective means of measuring how much the disease affects this population provides insights into targeted management interventions^21,22^. Both pharmacologic and non-pharmacologic interventions can enhance overall health and reduce poor health outcomes, and are recommended as an integral part of disease management strategies^23,24^. A systematic review and meta-analysis of randomized controlled trial showed significant overall HRQoL improvement after conducting psychosocial interventions for people with HF^25^. In Ethiopia, the government developed a comprehensive guideline for clinical management and care for major non-communicable diseases (NCDs) including HF. This is a major initiative aiming to reduce the increasing burden of NCDs in Ethiopia^26^.

Heart Failure is a major problem with high prevalence; however, few studies have investigated the impact of HF on HRQoL, especially in Africa. The available data indicates that poor quality of life in people with HF is a challenging problem in Ethiopia^27^. Consequently, this study sought to assess HRQoL and its influencing factors in people with HF based on the revised Wilson and Cleary model of HRQoL. Measuring HRQoL provides information about an individual’s overall health status and the impact of health interventions^28^. The findings from this study will inform healthcare policymakers on effective ways to improve care for this population in Ethiopia.

## Methods

### Study area and period

The study was conducted at the cardiac outpatient clinics of two tertiary level public hospitals in Addis Ababa, the capital of Ethiopia. St. Paul's Hospital Millennium Medical College and St. Peter Specialised Hospital are government-owned hospitals in Addis Ababa. They are Ethiopia’s largest tertiary-level specialist and teaching hospitals, providing referral services for people from all around the country. Currently, the two hospitals collaborate to provide care for patients with cardiac conditions. Each hospital had a weekly average of 30 people with HF coming to visit outpatient clinics for their routine follow-up care.

The survey was conducted between 21 Nov 2022 to 22 Jan 2023.

### Study design

A hospital-based survey using a cross-sectional study design was conducted.

### Theoretical framework

**Figure 1:**
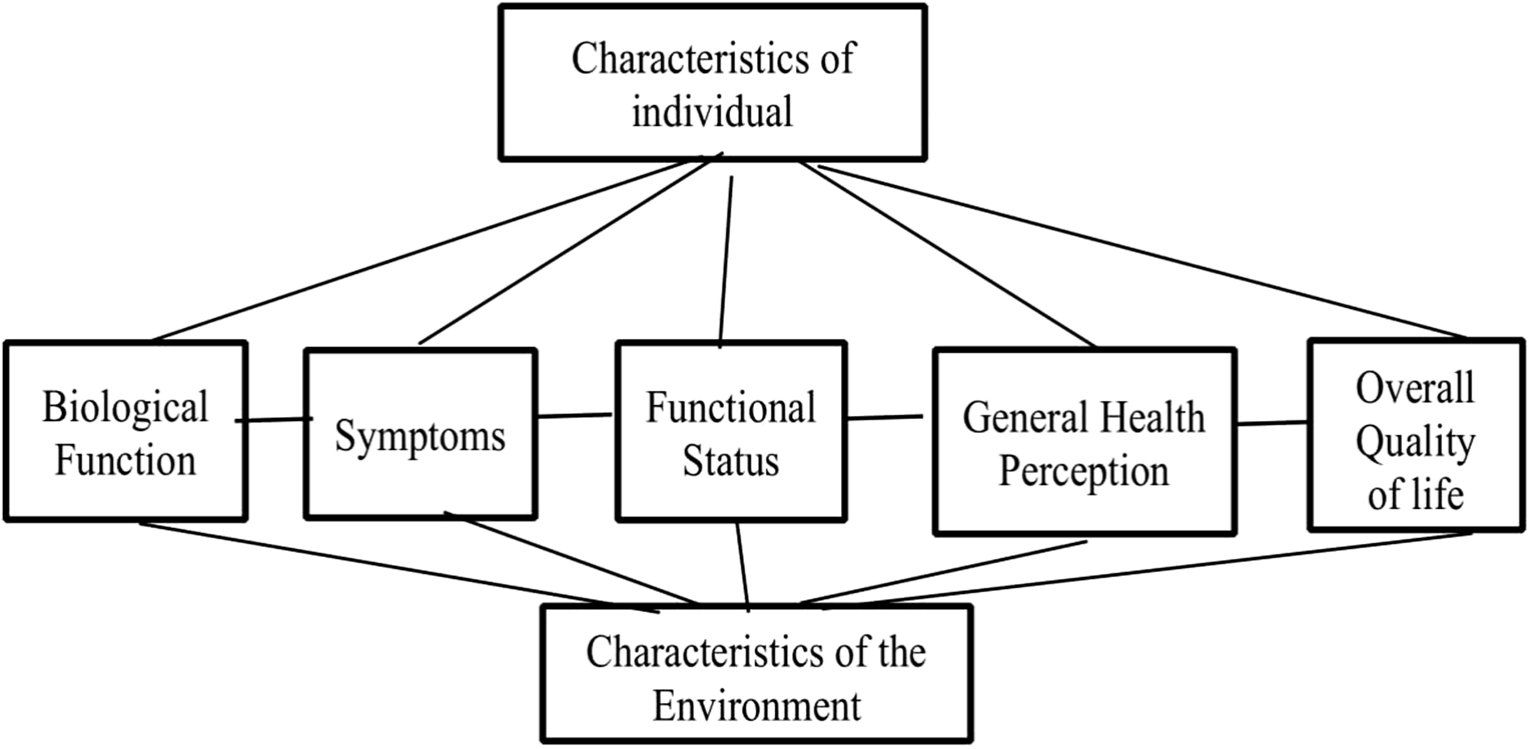
Revised Wilson and Cleary model of HRQoL^13^.

This study was guided by the revised version of Wilson and Cleary model of HRQoL^13^. This model provides a clear understanding of variables influencing HRQoL and clarify the relationships among them to guide quality of life research^29^. According to this model, HRQoL as the outcome variable is influenced by the characteristics of the individual, biological function, symptoms, functional status, characteristics of the environment, and general health perception (Fig. 1). It is the most widely used and the recommended model for HRQoL research^30,31^. This model, in the context of Ethiopia, provides a significant framework for assessing HRQoL that is adapted to the unique healthcare challenges of the country such as limited resources, poor healthcare access and diverse socioeconomic conditions that influence health outcomes^32^. By applying this model within this context, we identified factors affecting HRQoL of people with HF in Ethiopia.

### Eligibility criteria and ethics

The following inclusion criteria were considered: 1) adults aged over 18 years of age, 2) diagnosed with heart failure (clinically using Framingham criteria or Echocardiography), 3) receiving follow-up care at the outpatient cardiac clinic for at least three months. The study was approved by the University of Technology Sydney (UTS) Human Research Ethics Committees (UTS HREC REF NO. ETH21-6739). Local ethical approval was obtained from the institutional review board (IRB) of each hospital where data collection took place. All methods were performed in accordance with the relevant guidelines and regulations.

### Sample size and sampling procedure

The sample size was determined using a single proportion formula for finite population, N= (Zα/2)^2^*P (1 − P)^2^/D^2^)^33^, with the assumption of a 95% confidence interval, marginal error (d) of 5.0 % and 51.8% prevalence (P) of low HRQoL among people with HF in Northwest Ethiopia^34^. N= {(1.96)^2^*0.518 (1-0.518)}/0.05^2^=383 people with HF.

Consecutive sampling technique was used, and all eligible people with HF with an appointment at the outpatient cardiac clinics were invited to participate in the study. Participants who met the inclusion criteria and agreed to provide informed consent were included in this study until the required sample size was achieved.

### Variables

#### Dependent variable

- Health-related quality of life

#### Independent variables

- Characteristics of the individual
  - Age, sex, educational status, marital status, health insurance
- Biologic function
  - Family history of heart failure, comorbidities
- Symptoms
  - Depression
- Functional status
  - The severity of heart failure: New York Heart Association (NYHA) class
- Characteristics of the environment
  - Social support, residence, employment status, duration of illness, history of hospitalisations, number of medications taken each day.
- Overall health perception

### Operational definitions and definitions of terms

*Adult*: 18 years and over.

*Heart failure*: inability of the heart to effectively pump blood as evidenced by either signs and symptoms based on Framingham criteria or reduced ejection fraction (<40%)^35^^,^^36^.

*Health-related quality of life*: individuals’ perceptions and reactions to the impact of a disease or its treatment on their daily lives. It encompasses many dimensions of life, including physical, functional, emotional, and mental health^37^. Health-related quality of life of people with HF was measured by the Minnesota Living with Heart Failure Questionnaire (MLHFQ). This tool has 21 items or questions that asks participants how their disease has affected their life in the last month. Each question is coded using a six-point Likert-type scale that ranges from 0 (no effect) to 5 (very much effect). These scales are then added together to create a composite score that indicates the participant's overall HRQoL. The scores range from 0 to 105 with the higher composite scores represents the poorer quality of life. It gives a total score as well as scores on two dimensions: physical (from 2 to 7, 12, and 13, range, 0–40) and emotional (from 17 to 21, range, 0–25). The remaining eight questions (1, 8, 9, 10, 11, 14, 15, and 16) are only considered to calculate the total composite score^38–40^. It is a gold standard and widely used disease-specific quality of life measurement instrument that has been validated for use in many countries^20,41–45^.

*Depression*: feelings of unhappiness and lack of interest in daily activities with symptoms for at least two weeks based on DSM-5 diagnostic criteria^46^. Depression was measured using the patient health questionnaire (PHQ-9). This is a 9-item tool based on DSM-IV criteria that can be scored from 0 (not at all) to 3 (nearly every day). Total scores range from 0 to 27, with higher scores indicating a higher likelihood of depression. Each item requires participants to rate the frequency of a depressive symptom experienced in the two weeks prior to assessment. PHQ-9 is a reliable and valid instrument for detecting major depressive disorder among Ethiopian adults with chronic conditions in outpatient settings^47,48^.

The level of social support was measured using the Oslo Social Support Scale (OSSS-3). This scale consists of three items that asks about numbers of close confidants, sense of concern from other people, and relationships with neighbours, with a focus on the availability of practical help. The scores range from 3-14, with high values representing strong levels and low values representing weak levels of social support^49^. It has good construct and predictive validity and good internal consistency with a Cronbach alpha of 0.91^50^.

*General health perception*: a representation of all health concepts together which determined overall satisfaction with life using one global question that asks the respondent to rate their overall health on a Likert scale as "excellent", "very good", "fair" or "poor"^13^.

*The severity of heart failure*: New York Heart Association criteria (NYHA) is used to classify the severity of heart failure as Class I: no limitation during ordinary activity, Class II: slight limitation during ordinary activity, Class III: marked limitation of normal activities without symptoms at rest, Class IV: unable to undertake physical activity without symptoms and symptoms may be resent at rest^51^.

### Data collection procedure and instruments

The questionnaire was adapted from different literature and validated tools, and the process involved translation, cultural adaptation, and validation, which involved professional and language experts and pre-testing. These adaptations were made to ensure that the questionnaire was suitable for the cultural and linguistic context of Ethiopia. The questionnaire consisted of four parts: The first part related to sociodemographic and clinical related characteristics; the second part dealt with social support, the third part measured depression and the final section evaluated HRQoL. People with HF who met the eligibility criteria were contacted by trained research assistants (RAs) during their routine follow-up visit to the outpatient cardiac clinic of each hospital. RAs collected the data in person using an interviewer-administered questionnaire.

The sociodemographic and clinical characteristics were assessed using a 19-item questionnaire. This questionnaire collected information about age, sex, marital status, employment status, residence, educational level, health insurance, family history of HF, hospitalisation history, comorbidities, duration of illness, NYHA class, number medication taken each day and general health perception. The Oslo Social Support Scale (OSSS-3) was used to measure the level of social support and depression was measured using the patient health questionnaire (PHQ-9). Finally, the Minnesota Living with Heart Failure Questionnaire (MLHFQ) was used to measure the health-related quality of life of participants.

To ensure the quality of data, training on data collection procedure was provided to the research assistants. The collected data were checked for completeness and clarity daily. Two supervisors supervised the data collection process.

### Data management and analysis

Collected data were cleaned and entered into Epi-Data version 3.1 and exported to STATA Version 17 for analysis^52^. Descriptive analyses were performed to describe the socio-demographic and clinical characteristics of the participants, the OSSS-3, the PHQ-9, and the MLHFQ scores. A linear regression model was fitted to assess the association between independent variables and dependent variable. First, simple linear regression was performed to test the association between each independent variable with the dependent variable. All independent variables with a p-value of less than 0.25 on the simple linear regression analysis were eligible for further analysis in the multiple linear regression model. The assumptions of linear regression such as normality, linearity, independence, and homoscedasticity were checked. The results are presented in text, tables and graphs based on the types of data.

### Ethics approval and consent to participate

The study was approved by the University of Technology Sydney (UTS) Human Research Ethics Committees (UTS HREC REF NO. ETH21-6739), and local Ethical approval latter to conduct the study was obtained from the institutional review board (IRB) of each hospital where the data collection took place. The purpose, risk and benefit of the study were explained to the participants before conducting data collection. Written informed consent was obtained from participants who agreed to participate, and they were assured of the anonymity and confidentiality of their personal information. The data were collected in a quite area at the hospital.

## Results

### Sociodemographic and clinical characteristics

Three hundred and eighty-three (383) people with heart failure participated in this study with a response rate of 100%. Of these, 199 (51.96%) were female and 184 (48.04%) were male. The mean age of the participants was 55.1 (± 15.38) years. From all participants, 97 (25.33%) came from rural areas, and 196 (51.17%) were married. A total of 114 (29.77%) participants did not have any formal education. The Ethiopian Community Health Insurance (CHI) was held by 276 (72.06%) participants and 132 (34.46%) had a history of recent hospitalisation. Concerning comorbidities, 169 (44.13%) and 80 (20.89%) participants had hypertension and diabetes mellitus, respectively. The participants’ length of time since HF diagnosis ranged from 3 months to 22 years, with a mean of 4.80 years. Their mean social support and depression scores were 8.98 ±2.94 and 11.02 ±6.14, respectively. In the study, 100 (26.11%) participants had poor general health perception. Other sociodemographic and clinical characteristics are presented in Table 1.

**Table 1:** Sociodemographic and clinical characteristics of people with HF in Ethiopia, 2023 (n=383).

Variables	Category	N (%) or mean ± SD
Age (in years)	Mean score	55.1 ± 15.38
Sex	Male	184 (48.04)
	Female	199 (51.96)
Marital status	Single	65 (16.97)
	Married	196 (51.17)
	Divorced	33 (8.62)
	Widowed	69 (18.02)
	Separated	20 (5.22)
Employment status	Employed	184 (48.04)
	Unemployed	199 (51.96)
Residence	Urban	286 (74.67)
	Rural	97 (25.33)
Educational level	Uneducated	114 (29.77)
	Primary education	108 (28.20)
	Secondary education	87 (22.72)
	College and above	74 (19.32)
Health insurance	Yes	276 (72.06)
	No	107 (27.94)
Family history of heart failure	No	336 (87.73)
	Yes	47 (12.27)
History of hospitalization in the last 12 months	No	251 (65.54)
	Yes	132 (34.46)
Social support scores	Mean score	8.98±2.94
Comorbidities		
Hypertension	Yes	169 (44.13)
Diabetes	Yes	80 (20.89)
Kidney disease	Yes	29 (7.57)
COPD and asthma	Yes	11 (2.87)
Cancer	Yes	3 (0.78)
HIV/AIDS	Yes	19 (4.96)
Duration of illness (in years)	Mean score	4.80±4.65
Number pills taken each day	Mean score	3.98±2.01
NYHA class	Class I	135 (35.25)
	Class II	117 (30.55)
	Class III	93 (24.28)
	Class IV	38 (9.92)
General health perception	Excellent	32 (8.36)
	Very good	102 (26.63)
	Fair	149 (38.90)
	Poor	100 (26.11)
Depression scores	Mean score	11.02±6.14

### Health-related quality of life of people with heart failure

In this study, HRQoL of people with HF was assessed with MLHFQ. The mean of total MLHFQ score was 48.03±19.73, indicating a poor HRQoL among the study participants. Higher scores on the MLHFQ represent poorer quality of life. The subscale scores of MLHFQ were found to be 21.03±8.46 for physical scale and 10.12±6.26 for emotional scale (Table 2).

**Table 2:** Health related quality of life scores of people with HF in Ethiopia, 2023 (n=383).

MLHFQ scores	Possible range	Observed range	Mean + SD
Physical dimension	0–40	0–36	21.03±8.46
Emotional dimension	0–25	0–25	10.12±6.26
Total score	0–105	7–89	48.03±19.73

*MLHFQ* Minnesota Living with Heart Failure Questionnaire.

**Figure 2:**
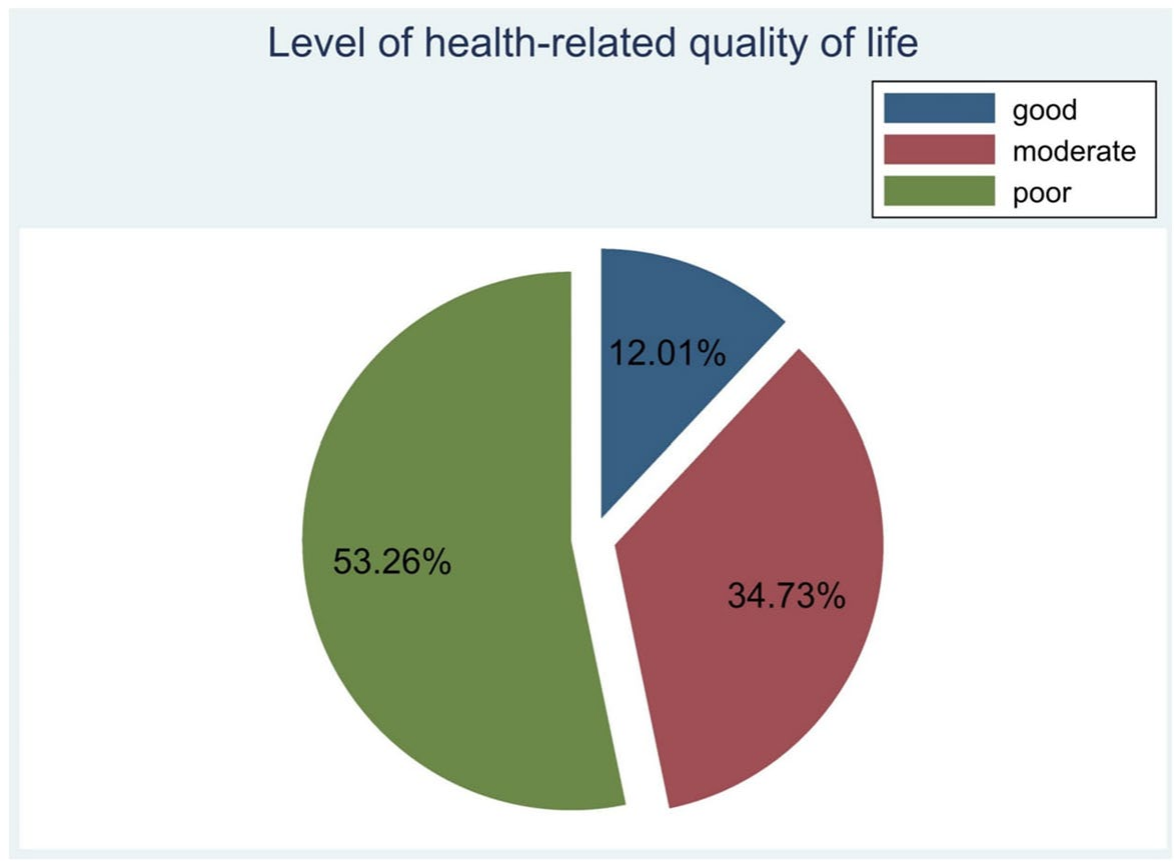
The level of HRQoL among people with heart failure in Ethiopia, 2023.

The MLHFQ score was used to determine the level of HRQoL as people who score less than 24 labelled as having (good) HRQOL, 24–45 (moderate), and greater than 45 as (poor) HRQOL^53,54^. The analysis revealed that 204 (53.26%) participants had a poor HRQoL (Fig. 2).

### Factors influencing the HRQoL of people with heart failure

Simple linear regression analysis demonstrated that variables such as age, sex, marital status, employment status, educational level, hospitalisation history, hypertension, diabetes mellitus, Acquired Immune Deficiency Syndrome (AIDS), New York Heart association (NYMA) class, social support scores, depression scores, and general health perception were factors associated with MLHFQ score at p<0.25 and were eligible for multiple linear regression. The multiple linear regression model revealed that only age, employment status, DM comorbidity, social support scores, NYHA class, depression scores, and general health perception were significantly associated with overall HRQoL of people with heart failure at p≤0.05. These variables explain 70% of variation of the overall MLHFQ scores.

Each extra year of age of participants predicts an increase in MLHFQ score by 0.12 (β = 0.12, CI (0.11, 0.28), p = 0.001). People who were unemployed scored an average 2.73-point higher MLHFQ score than employed people (β= 2.73, CI (0.22, 5.24), p = 0.033). A 1-unit increase in Oslo social support score corresponded to a 1.48-unit decrease in MLHFQ score (β = − 1.48, CI = (− 1.93, − 1.03), p=0.001). This means that strong social support is positively associated with HRQoL. People with diabetes scored an average 4.47-point higher MLHFQ score than those without diabetes (β = 4.47, CI (1.41, 7.54), p = 0.004). A 1 unit increase in the PHQ-9 score was associated with an increase in MLHFQ score by 1.744 (β= 1.74, CI (1.52, 1.96), p=0.001). Compared to participants with NYHA class I, those with class III (β=4.621, CI (1.50, 7.74), p=0.004) and IV (β=7.955, CI (3.66, 12.25), p=0.001) had a significantly higher HRQoL score, indicating poorer HRQoL as physical limitation or severity increases. The detailed result of multiple linear regression analysis is presented in Table 3.

**Table 3:** Multiple linear regression model for factors influencing the HRQoL of people with HF in Ethiopia, 2023 (n=383).

Variables	Β	SE	P-value	95% CI
Age	0.120	0.045	0.001*	(0.112, 0.287)
Sex				
Female	0.433	1.272	0.734	(−2.878, 2.935)
Marital status				
Single	2.614	1.608	0.105	(−0.548, 5.777)
Divorced	0.664	2.089	0.751	(−3.444, 4.773)
Widowed	−1.755	1.726	0.310	(−5.150, 1.639)
Separated	1.126	2.567	0.661	(−3.922, 6.174)
Employment status				
Unemployed	2.733	1.277	0.033*	(0.223, 5.244)
Educational level				
Primary	1.416	1.548	0.361	(−1.627, 4.460)
Secondary	1.782	1.708	0.298	(−1.578, 5.141)
College and above	1.828	1.826	0.317	(−1.763, 5.420)
Hospitalization history				
Yes	0.680	1.235	0.582	(−1.749, 3.108)
Hypertension				
Yes	0.906	1.203	0.452	(−1.459, 3.271)
Diabetes mellitus				
Yes	4.472	1.559	0.004*	(1.407, 7.537)
HIV/AIDS				
Yes	5.722	2.670	0.314	(−2.587, 8.031)
Social support scores	−1.481	0.228	0.001*	(−1.929, −1.032)
NYHA class				
Class II	1.006	1.417	0.478	(−1.781, 3.793)
Class III	4.621	1.586	0.004*	(1.502, 7.741)
Class IV	7.955	2.182	0.001*	(3.664, 12.245)
Depression scores	1.744	0.112	0.001*	(1.524, 1.965)
General health perception				
Very Good	3.914	2.247	0.082	(−0.504, 8.332)
Fair	4.098	2.204	0.064	(−0.237, 8.432)
Poor	4.940	2.349	0.036*	(0.321, 9.559)

Model statistics: R^2^ = 0.7023, F (22, 360) = 42.58, P < 0.001.

Reference category: Male, Married, uneducated, employed, NYHA “class I”, “Excellent” health perception, “No” for (hospitalisation history, hypertension, diabetes, HIV/AIDS).

β standardized beta coefficients, *SE* standard error, *CI* confidence interval.

*Statistically significant (p < 0.05).

## Discussion

Health related quality of life (HRQoL) is a critically important clinical outcome for people with HF^55^. This study investigated the HRQoL of people with heart failure and examined its influencing factors using the revised Wilson-Cleary HRQoL model. In this study, 53.26% (95%CI: 48.12, 58.35) of participants had poor HRQoL. This finding is consistent with the result of previous studies conducted in low-middle income countries^34,56,57^. The mean of total MLHFQ scores in the current study was 48.03 (95%CI: 46.04, 50.01), which indicates that people with HF have poor physical, emotional, and overall HRQoL. This finding is consistent with the previous similar studies that have shown the negative impact of HF on the physical, emotional and the general aspects of HRQoL^34,38,57^. The mean total MLHFQ score of the current study was higher than similar studies conducted in Brazil^58–60^, China^61^, Myanmar^1^, South Korea^16,62^ and Thailand^17^. The higher MLHFQ score in our population could be due to the cohort age as most participants were aged 40-69 years and had higher rate of depression than reported in studies from countries. Conversely, the mean of total MLHFQ score in this study was lower than studies conducted in Colombia^63^, Serbia^14,64^, Georgia^65^, and South Korea^66^. This difference might be due to variation in methodological approaches, sample size, socioeconomic characteristics, and study settings.

The current study identified various sociodemographic and clinical factors influencing the HRQoL of people with HF. Factors such as higher age, unemployment, poor social support, comorbid depression, diabetic comorbidity, higher NYHA class, and poor health perception were significantly associated with low HRQoL in people with HF, after controlling for known confounders such as gender, hospitalisation history, marital status, educational level, hypertension, and HIV/AIDS.

Sociodemographic factors such as age and employment had a strong influence on HRQoL, but gender had a weak influence. Increasing age was significantly associated with an increased MLHFQ score. This was supported by similar studies that reported a decrease in the HRQoL as people aged^16,67^. This finding can be attributed to the fact that older individuals experience more physical limitations and psychological problems^68^. A significant negative association between age and HRQoL was observed in other studies; the lower the age, the worse the quality of life^58,60^. According to the results of previous studies^16,57,69^, gender was a significant predictor of poor HRQoL, with women reporting significantly lower quality of life than men. Ahmeti et al. reported that females had poorer quality of life than males with HF^38^. However, there was no association between gender and HRQoL in the current study, which is consistent with Erceg et al. (2013) findings^70^. Regarding employment, people who were unemployed had a lower HRQoL than employed people. This finding is supported by previous studies^16,70,71^. This may be attributed to unemployed individuals often having a lower economic status and experience financial hardship due to medication and other health costs, which negatively impact management of HF and reduce their HRQoL^69^.

Another factor which influenced HRQoL in people with HF was social support. A strong social support network was associated with a better HRQoL. A similar study by Barutcu , et al. (2013) showed that higher levels of social support was significantly associated with better HRQoL in people with HF^72^. In addition, Soleimani et al. found that people with higher levels of social support had significantly improved HRQoL^73^. Other studies showed that social support improve HRQoL indirectly by enhancing self-care^66,74^. This could be because strong social support plays a critical role in helping individuals with emotional support that can help them to cope with the emotional burden of their condition and protect them from harmful outcomes, consequently improving HRQoL^66,75^.

In the current study, comorbid depression and diabetes mellitus predicted lower HRQoL with a significant association between depression and HRQoL. This finding is consistent with previous studies that reported a strong association between depression and HRQoL^14,65,70,76–80^. Comorbid depression negatively influences an individual's psychosocial wellbeing and affect selfcare practices and treatment adherence^81,82^. Similarly, a significant influence of comorbid diabetes mellitus on the overall HRQoL was observed. The same finding has been reported in previous studies^83–85^. People with diabetes who have poor glycaemic control and associated complications are more likely to experience various health problems that significantly lower HRQoL^86^. In addition, non-cardiovascular comorbidities accelerates disease progression and increases risk of rehospitalisation which can lead to additional socioeconomic burden, ultimately reducing HRQoL^80^.

In the present study, people with NYHA class III and IV were associated with poor HRQoL. This finding has been reported in many previous studies^70,87,88^. The possible justification for impaired HRQoL being correlated with higher NYHA class could be because the ability to carry out daily activities decreases as the severity of the disease increase, which negatively affects quality of life^54^.

## Limitations and suggestions for future research

The findings of this study have implications for clinical practice. Determining the quality of life of people with HF provides information about the impact of HF on overall perceived well-being. A better understanding of factors influencing HRQoL may help health care professionals to identify effective interventions to improve individuals’ HRQoL. This study has several limitations that should be considered when interpreting its results. First, its cross-sectional nature limits its ability to establish cause-and-effect relationships. Therefore, longitudinal studies in this domain are recommended to investigate causal relationships. Second, the use of a self-report questionnaire to collect data in this study could lead to information bias, and participants could be influenced by recall bias or the Hawthorn effect^89^. Finally, the influence of critical factors from the Wilson and Cleary model, such as selfcare practice, medication adherence and HF knowledge, were not evaluated in this study. Future research should evaluate these variables to gain a deeper understanding of their influence.

## Conclusion

This study reported the impact of HF on the HRQoL in an Ethiopian cohort. The results revealed that more than half of all people living with HF in Ethiopia had a poor HRQoL, as demonstrated by the mean score of total MLHFQ and its subscales. Factors such as age, employment status, depression, social support, diabetic comorbidity, NYHA class, and health perception were significant in influencing the HRQoL of people with HF. Future interventional studies are recommended with the aim of improving the HRQoL of these population.

## Data Availability

All the data analysed in this study are available from the corresponding author upon a reasonable request.

## Abbreviations

CI: Confidence interval
CVD: Cardiovascular disease
HF: Heart failure
HRQoL: Health-related quality of life
MLHFQ: Minnesota living with heart failure questionnaire
NYHA: New York heart association
NCDs: Non-communicable diseases
OSSS-3: Oslo social support scale
PHQ-9: Patient health questionnaire
SE: Standard error
